# Clinical Electrophysiology and Mathematical Modeling for Precision Diagnosis of Infertility

**DOI:** 10.3390/biomedicines13020250

**Published:** 2025-01-21

**Authors:** Fernanda Carvalho Cavalari, Paola Sulis Mendes, Bruna Antunes Zaniboni, Carine Royer, Bárbara Ogliari Martins Taques, Karina Cesca, Marcela Aragón, Fátima Regina Mena Barreto Silva

**Affiliations:** 1 Instituto de Bioeletricidade Celular (IBIOCEL): Ciência & Saúde, Departamento de Bioquímica, Centro de Ciências Biológicas, Universidade Federal de Santa Catarina, Campus Universitário, Rua João Pio Duarte Silva, 241, Sala G301, Florianópolis 88037-000, SC, Brazil; fernanda.labenex@gmail.com (F.C.C.); pasulis@hotmail.com (P.S.M.); brunaantunesz@outlook.com (B.A.Z.); cariroyer@unb.br (C.R.); btaques@ifsc.edu.br (B.O.M.T.); karinacesca@gmail.com (K.C.); dmaragonn@unal.edu.co (M.A.); 2 Laboratório de Farmacologia Molecular, Universidade de Brasília, Brasília 70900-910, DF, Brazil; 3 Faculdade de Ciências e Tecnologias em Saúde, Universidade de Brasília, Brasília 70900-910, DF, Brazil; 4 Instituto Federal de Santa Catarina, Joinville, Santa Catarina, Av. Sete de Setembro, 3165, Curitiba 80230-901, PR, Brazil; 5 Departamento de Engenharia Química, Centro de Tecnologia, Universidade Federal de Santa Catarina, Florianópolis 88038-000, SC, Brazil; 6 Departamento de Farmácia, Universidad Nacional de Colombia, Av. Carrera 30 # 45-03 Edif 450, Bogotá 111321, Colombia

**Keywords:** electrophysiology, mathematical model, precision medicine, signaling pathway, infertility, drug development

## Abstract

How can cellular electrophysiology measurements and mathematical modeling of ionic channels help to identify pivotal targets in disease-related cell signaling? The purpose of this review is to highlight the advantages and disadvantages of using both of these complementary techniques to determine molecular targets that may be structurally or functionally altered in a specific disease. In addition, both electrophysiology measurements and mathematical modeling may improve coordinated drug development, accelerate the prediction of new drugs, and facilitate repositioning of pharmacological agents. This review focuses on the data obtained from electrophysiology and mathematical model approaches, including intracellular recording, cellular patch clamp measurements, and the Hodgkin and Huxley equation, as key precision methodologies. To this end, seminiferous tubules, the Sertoli cell line (TM4), and/or primary cultures of Sertoli cells were used to explore the role of follicle-stimulating hormone (FSH), thyroid hormones, retinol, testosterone, and 1,25(OH)_2_ vitamin D_3_ in the coordinated activation or inhibition of ionic channels essential for male fertility. Based on the discussed data, Sertoli cells precisely regulate their biological activity by coordinating channel activity according to the hormonal environment and the nutritional requirements required for germ cell development.

## 1. Introduction

Despite the cellular heterogeneity within the testis, Sertoli, Leydig, peritubular, and germ cells can function independently, according to their specific roles, while also cooperating dynamically through intacrine, paracrine, and/or autocrine actions. This guarantees spermatogenesis and steroidogenesis under the control of gonadotrophins, androgens, and several factors locally produced, such as estrogens [1,2,3,4,5,6,7]. Steroidogenesis in the testis occurs mainly in the Leydig cells, particularly the synthesis of testosterone, which can be irreversibly converted into estradiol in the Sertoli cells by a protein complex of cytochrome P450 aromatase [1].

The spermatogenesis wave is influenced by different factors, such as hormonal, environmental, and nutritional factors, as well as genetic and epigenetic diseases [8,9].

The main physiological role of the somatic Sertoli cells is to maintain spermatogenesis through the blood–testis barrier by providing the mechanical and nutritional support to neighboring germ cells in order to create a suitable immunological and endocrine microenvironment [10,11,12,13]. Therefore, the number and health of the Sertoli cells at the prepubertal stage is determinant for spermatozoa production to guarantee fertility throughout adult life [14,15,16]. The secretory activity of Sertoli cells relies on ionic changes that are triggered by voltage-dependent calcium channel, potassium channel, and chloride channel activities to promote exocytosis of a fluid rich in ions, nutrients, proteins, and growth hormones to germ cells to support the progression of the spermatogenic wave [17,18]. On the other hand, the dysfunction of Sertoli cells may be implicated in a low concentration and scarce sperm count, resulting in infertility [19,20].

## 2. Electrophysiology and Mathematical Models

Electrophysiology is a definitive and powerful approach for investigating ionic channel-related diseases with high precision and celerity. This methodology offers the possibility of recording the electrical behavior of cells through a diversity of technical conformations within recording modes, such as intracellular recording and patch clamp techniques (cell attached, whole cell, excised path) to provide unprecedented insights into cellular activity [20,21,22,23,24]. One of the properties of electrophysiology recordings is signal processing and recording at high speed and in real time for a variety of linear and non-linear operations. These recordings can accurately translate the channel activity that is related to a critical cell function in health or disease [25,26,27]. The membrane potential is a physical chemical variable that may trigger cell activation processes, resulting in secretion (exocytosis), plasma membrane protein activation (opening/closing ionic channel), cell contraction (cytoskeleton), pumping, and plasma membrane enzyme activation [28]. With reference to these parameters, key data were selected to explore the hormonal regulation of Sertoli cell secretion during the determinant window of testis maturation at the pre-pubertal and pubertal phases [29].

Mathematical analysis represents an important tool for unraveling mechanisms that drive biological systems and can be applied to electrophysiology using the patch clamp method to obtain the curves used in theoretical models [27,28,29,30,31]. The mathematical model for predicting signals caused by conformational changes in ionic channels due to changes in ionic permeabilities, cellular ATP, and other quantities is of great importance for understanding cellular behavior in the presence of thyroid hormones, FSH, and testosterone, among others [29,32,33,34,35,36].

## 3. Follicle-Stimulating Hormone Alters the Plasma Membrane Potential in Sertoli Cells

Follicle-stimulating hormone (FSH) is a glycoprotein secreted by adenohypophysis that binds to a pleiotropic receptor in the testis exclusively at the plasma membrane of Sertoli cells [37]. The signal transduction of FSH relies on the genomic responses for Sertoli cell proliferation and the maintenance, differentiation, and apoptosis of spermatogonia. Furthermore, FSH plays a central role in the development and function of the testis via cAMP/PKA, PI3K/Akt, and ERK/MAPK signaling for genomic responses [33,34,35]. Sertoli cells exhibit a fine diversity of electrophysiological properties, depending on the phase of testicular maturation, the activity of the spermatogenic wave, and the influence of the hormonal or chemical environment. FSH also exhibits a complementary and efficient route of signal transduction, known as non-classical or rapid response pathways (seconds to minutes), which regulate the electrolytic homeostasis and coordinate the secretory activities of Sertoli cells to the germ cells that may support the ongoing spermatogenesis [21,29]. As such, electrophysiological recordings demonstrate that FSH produces immediate hyperpolarization, followed by depolarization, in Sertoli cells in the seminiferous tubules of the immature rat testis. One of the intracellular pathways through which FSH influences ionic homeostasis is via the opening or closing of plasma membrane ionic channels in the Sertoli cells, which can alter the intracellular ionic calcium concentration to activate a number of cellular functions, such as exocytosis [21,22]. The advantage of using this technique for such observations is its ability to precisely delineate the ionic mechanism coordinated by FSH (rapid responses/seconds) during amino acid transport. This mechanism is independent of those mediated by the serpentine receptors coupled to G-proteins in the plasma membrane, making this a valuable tool for studying the amino acid-related diseases associated with male infertility [37]. However, this raises the question: how this model aligns with human physiology? To complement these studies, with the popularization of mathematical modeling software, we developed the first mathematical model for potassium channel activity, based on recordings of electrical currents induced by K^+^ efflux in voltage-sensitive potassium, K_v_ (the currents responsible for rapid responses in Sertoli cells), obtained using the patch clamp method [32]. This model determined the membrane potential changes following the administration of the FSH hormone (from −44 ± 0.5 to −52 ± 0.2 mV) in the cell and demonstrated great similarity with the in vitro electrophysiological recordings obtained by Wassermann et al. [21]. The model uses potassium channel currents to obtain the equations demonstrated in the Hodgkin and Huxley model in 1952 [30]. The membrane potential changes caused by hormones produce an electrical stimulus input for the H-H model. Such changes are caused by the ATP-dependent Na^+^/K^+^ pump, which can be modeled by several methods. In this model, the equations of the Na^+^/ K^+^ enzymatic model described by Hernandez et al. [31] were applied to determine the relationship between the Na^+^/K^+^ pump and the membrane potential. Variations in hormone concentration administered to the cell caused changes in the ATP-dependent Na^+^/K^+^ pump current, opening the K*v* channels. The validation of this mathematical modeling for induced hormonal hyperpolarization was warranted, as the maximal error was about 3% compared with the simulated and experimental results for FSH plus verapamil, as disccused by Taques et al. [32].

Additionally, techniques such as those that use radiolabeled compounds, e.g., [1-^14^C]MeAIB and ^45^Ca^2+^ influx, associated with electrophysiology, have been able to characterize a plasma membrane target for FSH on amino acid transporters and calcium channels, signifying an important discovery related to hormonal rapid responses [21,38]. As such, this profile of multimodal electrophysiology can improve precision medicine approaches and be used to suggest molecular structures that interact perfectly with a therapeutic target.

## 4. Thyroid Hormones Act on Sertoli Cells by Activating Potassium Channels

Thyroid hormones, such as thyroxine (T_4_), 3,5,3′-L-triiodothyronine (T_3_), 3,3′,5′-triiodothyronine (rT_3_), and even the metabolite of rT_3_, O-(4-hydroxyphenyl)-3-3′-diiodo-L-tyrosine (T_2_), have defined roles in the testis, depending on the phase of testicular development. These hormones are critical during the postnatal period before the formation of the blood–testis barrier to control Sertoli cell proliferation and, therefore, the number of spermatozoa [39].

In the mid-1990s, following the discovery of various thyroid receptor isoforms, interest grew in the nongenomic effects of thyroid hormones mediated by cell surface receptors [40]. Using intracellular recordings, our laboratory reported that T_3_ and T_4_ act directly at the plasma membrane on amino acid accumulation and induce immediate hyperpolarization, mediated by potassium and calcium channels, in intact Sertoli cells in seminiferous tubules. In addition, the intracellular recording technique allowed us to distinguish the genomic and nongenomic effects of both of these thyroid hormones in Sertoli cells, as well as in other tissues [41,42]. The actions of thyroid hormones in Sertoli cells are also characterized by the absence of nuclear receptor activation, and are rather mediated by membrane-initiated mechanisms, known as non-classical hormone-binding elements, at the plasma membrane. In the whole immature rat testis, thyroxine interacts with the plasma membrane of Sertoli cells, opening potassium channels (K^+^-ATP and K^+^-Ca^2+^) and calcium-dependent chloride channels (Cl^−^-Ca^2+^), and hyperpolarizing the cells. This hyperpolarization triggers the opening of voltage-dependent Ca^2+^ channels, Ca^2+^ influxes, and depolarization, which then stimulates Na^+^-amino acid co-transport. Additionally, transient modulations in cytosolic Ca^2+^ involve protein kinase (PK) C, a pivotal protein that may regulate phosphorylation of plasma membrane ionic channels and/or support crosstalk with gene transcription [17]. As a whole, the ionic environment influences the synthesis and secretion of a reasonable number of proteins needed for germ cell development. Likewise, the bidirectional exchange of molecules between germ and Sertoli cells depends on the ionic involvement to support secretory activities [8]. In addition, the T_4_ integrin plasma membrane receptor activates amino acid uptake but does not mediate the calcium influx, cell secretion, or the nuclear effects of the hormone.

As such, these data suggest that the physiological responses of Sertoli cells to thyroid hormones are dependent upon continuous crosstalk for different signal transduction pathways to be accomplished. In the case of rT_3_, this molecule triggers rapid responses such as calcium influx and exocytosis, mediated by integrin receptors in the Sertoli cells. This suggests that different thyroid hormone metabolites act on specific binding sites at the plasma membrane to start distinct responses [43,44]. Building on these data, the new ionic target of rT_3_ at the plasma membrane of Sertoli cells was precisely identified by electrophysiology using whole-cell patch clamp techniques. Findings showed that rT_3_-induced calcium influx involves potassium efflux and selective activation of ionic calcium channels, and the Na^+^/K^+^ ATPase pump, all occurring within less than 5 min. These explicit nongenomic responses effectively coordinate exocytosis. Studies on T_2_ also merit attention, as its metabolite demonstrates a stimulatory effect on calcium influx in immature rat Sertoli cells. Given the very low concentrations of T_2_ (10^−15^ and 10^−12^ M) and the immediate maximum response (30 s), we conclude that its effect is plausible, as T_2_ is the final metabolite to be inactivated [22,43].

## 5. The Influence of Retinol on Calcium and Potassium Channels in Sertoli Cells

Retinol plays a known role in testicular functions by influencing spermatogenic processes. Excess retinol can cause spermatogenic lesions, while deficiency interrupts the ongoing spermatogenic wave and reduces cell secretion. Retinoids can act on Sertoli cells, germ cells, and Leydig cells, modifying the secretory activity of Sertoli cell functions, disturbing secretory activity, and decreasing cell proliferation and differentiation [45,46,47]. The rapid responses or non-classical mechanism of action of retinol in the Sertoli cells of immature rat testes was first reported in the 1990s by Loss and colleagues [48]. The authors demonstrated that the plasma membrane action of retinol in Sertoli cells was mediated by voltage-dependent calcium channels to regulate calcium influx and amino acid uptake. The classical intracellular recording showed an immediate oscillatory membrane potential response, followed by a significant reduction in membrane potential. These retinol events were associated with calcium influx and amino acid uptake studied in the whole testis, isolated Sertoli cells, and whole seminiferous tubules from the immature rat testis [47,48,49,50].

With regard to the vitamin A endocrine system, we demonstrated, using the patch clamp technique, that 10^−6^ M retinol after 5 min increased voltage-dependent potassium currents (Kv) in Sertoli cells. The inhibition of these responses by tetraethylammonium (TEA) confirmed a role of TEA-sensitive K^+^ channels in these effects. Additionally, using precise patch clamp techniques, we reported, for the first time, crosstalk between retinol and testosterone, as well as depolarization and activation of repolarization by K_v_ currents in Sertoli cells [49]. These ionic modulations play a physiological role in Sertoli cells, spermatogenesis, and male fertility via stimulation of secretory activities.

## 6. Testosterone Modulates Electrical Activities in Sertoli Cells

Using intracellular recording with the standard single-microelectrode technique, the roles of epitestosterone and testosterone in both the proliferative and non-proliferative phases of Sertoli cells were found to be similar but independent of the classical androgen receptor. In addition, epitestosterone, an 17α-epimer of testosterone, exhibits a rapid response that involves calcium and depolarization of Sertoli cells. Similar data were also obtained when recordings were carried out in ex vivo intact seminiferous tubules from the immature rat testis. In this case, electrophysiology served as useful tool to separate the plasma membrane effects of epitestosterone and testosterone from their genomic responses, which are mediated by classic androgen receptors [50,51].

Another advantage of using this technique is that it takes into consideration the fine architecture of Sertoli cells. The plasma membranes of Sertoli cells cover, or are the scaffolding of, seminiferous tubules. As described in the literature, Loss et al. [48] standardized a protocol for recording the membrane potential of Sertoli cells by crossing over the plasma membrane of myoid peritubular cells within enriched seminiferous tubules. Data collected from ex vivo and intact seminiferous tubules demonstrated a similar resting potential to that found in SCE seminiferous tubules, where SCE refers to testes without germ cells after embryos have been irradiated in utero with 1 gray of ^60^Co between 19 and 21 days of pregnancy.

In another electrophysiological recording conformation, whole-cell electrophysiology showed that both testosterone and retinol increased voltage-dependent potassium currents (Kv) in isolated Sertoli cells. These novel data suggested the occurrence of cross-talk between retinol and testosterone, triggered by a rapid response mediated by voltage-dependent calcium channels and culminating in a depolarization followed by a repolarization of Sertoli cells, thereby contributing to exocytosis and guaranteeing male fertility [49].

## 7. 1,α25(OH)_2_ Vitamin D_3_ Activates Chloride Channels and Exocytosis in Sertoli Cells

The active form of vitamin D, 1α,25(OH)_2_ vitamin D_3_ (1,25-D_3_), is a secosteroid hormone that affects biological functions in the organism, such as calcium and phosphate homeostasis, as well as the expression of proteins such as calcium-binding protein (CaBP), by non-genomic responses and genomic responses, respectively [52,53,54,55,56].The testis is one of 38 tissue types in which the vitamin D receptor (VDR) is expressed, and it also is expressed in Sertoli and germ cells [57,58]. The hormonal regulation of fluid and protein secretion by Sertoli cells is critical throughout the spermatogenic wave and involves ionic channel modulation. Surprisingly, 1,25-D_3_ is reported to trigger the activation of chloride channels in the Sertoli cell line, TM4, characterizing the precise regulation of the electrochemical gradient during the exocytosis process [18,59,60]. In Sertoli cells, the high K^+^ causes a strong depolarization followed by a repolarization caused by K^+^ efflux which is mediated by the activation of voltage-dependent K^+^ channels. In response to 1,25-D_3_, within 10 min of exposure, the efflux of K^+^ was blocked by TEA, an inhibitor of voltage-dependent K^+^-channels, indicating that secretory activity and repolarization of Sertoli cells occurred through the Ca^2+^ conventional exocytosis pathway. Although several channels orchestrate the stimulus for Sertoli cell secretion, the recovery of membrane potential is coordinated by different and select K^+^ ionic channels, such as ATP-dependent K^+^ channels, voltage-dependent K^+^ channels, and Ca^2+^-dependent K^+^ channels. Based on the precise measurement of ionic channel activity in the presence of 1,25-D_3_, we demonstrated, for the first time, the non-genomic action of this hormone in primary cultures of Sertoli cells from the immature rat testis, which was mediated by K^+^-current potentiation coupled to exocytosis. This effect suggests the occurrence of a Ca^2+^ influx followed by K^+^ efflux and repolarization, characterizing a functional role for 1,25-D_3_ in male fertility via stimulation of cell secretion.

## 8. Conclusions

**Figure 1:**
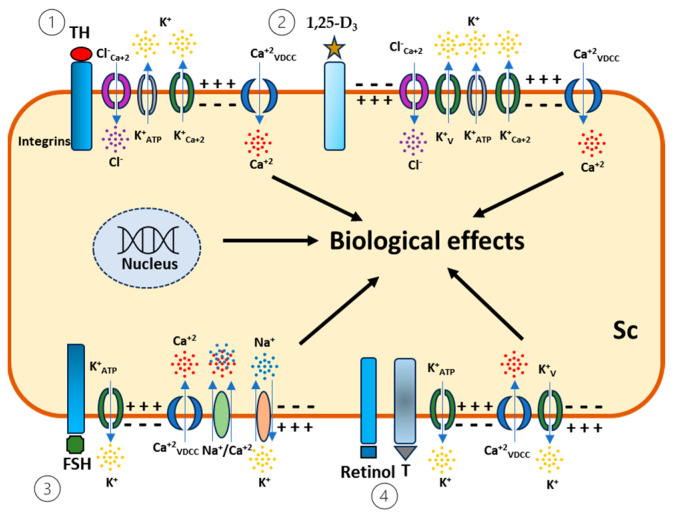
Precise ionic signaling mediated by hormones in Sertoli cells (Sc). (1) Thyroid hormone (TH) binds to the integrin receptor and opens calcium-dependent chloride channels (ClCa^2+^), inducing hyperpolarization in association with the activation of calcium-dependent potassium channels (K^+^-Ca^2+^) and ATP-dependent potassium channels (K^+^-ATP), which leads to the activation of voltage-dependent calcium channels (VDCC) and contributes to the secretory activities of Sertoli cells and the maturation of the testis. (2) 1,α25(OH)_2_ Vitamin D_3_ (1,25-D_3_) activates calcium-dependent chloride channels (ClCa^2+^), K^+^-Ca^2+^, K^+^-ATP, and voltage-dependent potassium channels (Kv) through plasma membrane receptors. This leads to the hyperpolarization and activation of VDCC, driving the downstream signaling of 1,25-D_3_ in the ongoing process of spermatogenesis. (3) Follicle-stimulating hormone (FSH) induces immediate hyperpolarization followed by depolarization in Sertoli cells through the activation of K^+^-ATP channels, VDCC, the Na^+^/Ca^2+^ exchanger, and Na^+^/K^+^-ATPase to trigger cellular functions such as exocytosis. (4) Retinol and testosterone bind to a specific plasma membrane receptor and promote rapid responses by crosstalk signaling involving the activation of VDCC, thereby triggering depolarization followed by repolarization, mediated by Kv currents in Sertoli cells, and stimulating secretory activities.

Based on the data discussed herein, the Sertoli cell precisely regulates its biological activity through the coordinated activity of channels, which are modulated according to the hormonal microenvironment, in order to maintain the nutritional requirements for germ cell development (Figure 1). Even with the availability and wealth of techniques to develop different experimental models, the translation of data obtained from animals to humans is not directly applicable and maybe not reliable. Nonetheless, the repetition of these biological effects predicts a valuable signaling pathway to accelerate drug development.
